# The Effect of Epstein-Barr Virus Latent Membrane Protein 2 Expression on the Kinetics of Early B Cell Infection

**DOI:** 10.1371/journal.pone.0054010

**Published:** 2013-01-08

**Authors:** Laura R. Wasil, Monica J. Tomaszewski, Aki Hoji, David T. Rowe

**Affiliations:** Department of Infectious Diseases and Microbiology, Graduate School of Public Health, University of Pittsburgh, Pittsburgh, Pennsylvania, United States of America; University of Sussex, United Kingdom

## Abstract

Infection of human B cells with wild-type Epstein-Barr virus (EBV) *in vitro* leads to activation and proliferation that result in efficient production of lymphoblastoid cell lines (LCLs). Latent Membrane Protein 2 (LMP2) is expressed early after infection and previous research has suggested a possible role in this process. Therefore, we generated recombinant EBV with knockouts of either or both protein isoforms, LMP2A and LMP2B (Δ2A, Δ2B, Δ2A/Δ2B) to study the effect of LMP2 in early B cell infection. Infection of B cells with Δ2A and Δ2A/Δ2B viruses led to a marked decrease in activation and proliferation relative to wild-type (wt) viruses, and resulted in higher percentages of apoptotic B cells. Δ2B virus infection showed activation levels comparable to wt, but fewer numbers of proliferating B cells. Early B cell infection with wt, Δ2A and Δ2B viruses did not result in changes in latent gene expression, with the exception of elevated LMP2B transcript in Δ2A virus infection. Infection with Δ2A and Δ2B viruses did not affect viral latency, determined by changes in LMP1/Zebra expression following BCR stimulation. However, BCR stimulation of Δ2A/Δ2B cells resulted in decreased LMP1 expression, which suggests loss of stability in viral latency. Long-term outgrowth assays revealed that LMP2A, but not LMP2B, is critical for efficient long-term growth of B cells *in vitro*. The lowest levels of activation, proliferation, and LCL formation were observed when both isoforms were deleted. These results suggest that LMP2A appears to be critical for efficient activation, proliferation and survival of EBV-infected B cells at early times after infection, which impacts the efficient long-term growth of B cells in culture. In contrast, LMP2B did not appear to play a significant role in these processes, and long-term growth of infected B cells was not affected by the absence of this protein.

## Introduction

Epstein-Barr virus (EBV) is a human gammaherpesvirus that is estimated to infect 95% of the human adult population worldwide. EBV targets human primary B cells, leading to primary B cell proliferation and, ultimately, establishment of a lifelong, latent infection. Symptoms of EBV infection are typically mild and often indistinguishable from other mild common illnesses. However, EBV infection during adolescence or young adulthood can lead to infectious mononucleosis, for which symptoms include fever, sore throat, fatigue and swollen lymph glands. Previous studies have demonstrated correlations between EBV infection and the development of various lymphomas and carcinomas in both pediatric and adult settings, including Burkitt's lymphoma (BL), Hodgkin's lymphoma (HL) and nasopharyngeal carcinoma (NPC) [1], [2], [3]. In addition, EBV infection is thought to play a critical role in the development of proliferative diseases in immunosuppressed patients, such as post-transplant lymphoproliferative disorder (PTLD), where EBV-transformed B cells proliferate continuously in the absence of an effective T cell response [4], [5].

Infection of primary human B cells *in vitro* leads to the establishment of lymphoblastoid cell lines (LCL) [6], [7], which is an important model for studying the tumorigenic properties of EBV. EBV-infected B cells can demonstrate several different patterns (Latency 0, I, II, III) of EBV latent gene expression. Latency 0, in which no genes are expressed, is thought to be the state of the virus found in B cells in the blood of all healthy carriers. This ability of EBV to restrict expression of its genes allows the virus to persist *in vivo* within resting memory B cells for the lifetime of the host [8], [9]. Latency I and II, which characterize many virus-associated tumors, show expression of Epstein-Barr Nuclear Antigen 1 (EBNA1), LMP2A, EBV-encoded noncoding RNAs (EBERs) and the BamHI A rightward transcripts (BARTs). The Latency II program expresses these genes but also results in expression of all three latent membrane gene products (LMP1, LMP2A and LMP2B). In Latency III, all the above genes and 5 additional EBNAs are expressed [10]. Expression of most of the Latency III genes is required for the growth program, which is characterized by antigen encounter-like activation of resting B cells and induction of proliferation [11], [12], [13], [14], [15], [16], [17].

The introduction of infectious virions early in EBV infection is critical for the outgrowth of spontaneous LCLs [18], [19] because it allows the virus to spread within the B cell population to activate uninfected cells. The production of infectious EBV requires a switch from the viral Latency III program to the lytic cycle. This lytic switch can be affected by both endogenous and exogenous stimuli, and can be characterized by a sequential cascade of gene expression of immediate early, early, and late genes [20]. The EBV gene BZLF1 encodes the immediate early lytic transactivator Zebra, which is necessary to trigger lytic switch by driving expression of lytic genes while downregulating latent genes [21], [22], [23]. The expression of Zebra alone has been shown to initiate lytic switch in various cell types [24], [25], [26]. A variety of exogenous stimuli, such as protein kinase C agonists (phorbol esters), histone deacetylase inhibitors (n-butyrate) and B cell receptor (BCR) signal induction, have been shown to initiate the lytic cycle [27].

The LMP2 gene produces two isoforms (LMP2A and LMP2B) of a 12 transmembrane (TM)-containing membrane protein. Circularization of the EBV genome is required for expression of LMP2A and LMP2B because transcription crosses the fused terminal repeats. These transcripts utilize unique promoters and distinct initial exons to encode the different LMP2 isoforms [28], [29]. LMP2A exon 1 encodes an N-terminal cytoplasmic region, which contains an immunoreceptor tyrosine-based activation motif (ITAM) responsible for initiating a B cell receptor (BCR)-like signal [30], [31]. This signal allows LMP2A to supply EBV-infected B cells with a strong BCR-like survival signal [32], which accounts for the ability of LMP2A to protect BCR-negative B cells from apoptosis [33], [34], [35], as well as block signaling through the BCR that would lead to lytic reactivation [31], [36]. The BCR-like signal provided by LMP2A may also mimic an activation signal. LMP2A can stabilize β-catenin in epithelial cells through protein kinase C-mediated inhibition of glycogen synthase kinase-3 (GSK-3), a process also performed through activation of the BCR in B cells [37], [38]. Also, other studies have demonstrated that LMP2A expression in B cells resulted in activation of protein tyrosine kinases (PTKs) and calcium (Ca^2+^) fluxes that resembled responses initiated by an activated BCR [39]. The role of LMP2A in proliferation and transformation is less clear, with some studies claiming the protein plays no role in proliferation and transformation of B cells *in vitro* [40], [41], [42], while other studies have demonstrated an essential role in this process [43]. Interestingly, LMP2A expression in HaCaT epithelial cells induces morphological changes that coincide with increased proliferation and loss of differentiation markers and cell anchorage, demonstrating that LMP2A signaling induces epithelial cell transformation [44].

LMP2B exon 1 is noncoding, and, therefore, LMP2B lacks an N-terminal signaling domain. Transcription of LMP2B initiates at a bidirectional promoter that is shared with LMP1, a protein that is critical for B cell transformation *in vitro* [12], [13], [14]. The LMP2A and LMP2B transcripts are identical in exons 2–9, which encodes the 12 TMs and a C-terminal tail that is required for protein aggregation. Without a signaling domain, LMP2B by itself cannot initiate a BCR-like signal, but it localizes to intracellular regions in B cells that contain signaling proteins, such as LMP2A and CD19 [45]. Although no role has as yet been demonstrated for LMP2B in either the activation or proliferation of B cells, many EBV-related malignancies, such as HL, NPC and gastric carcinoma, express both LMP2 isoforms [46]. Previous studies suggest that LMP2A and LMP2B contribute to epithelial cell spreading and motility, and may contribute to epithelial cell transformation [47]. LMP2B has been implicated as a critical player in the switch from viral latency to lytic reactivation [48], [49].

In this work, we present an analysis of the role of the EBV proteins LMP2A and LMP2B in early B cell infection *in vitro*. Our analysis was performed using viruses deficient in LMP2A and/or LMP2B for infection of human B cells obtained from healthy donors in order to assess the roles of these proteins in the processes of activation, proliferation, and survival during early infection. In addition, we examined roles for LMP2A and LMP2B as regulators of latent gene expression and viral latency that could further explain differences in early infection kinetics. Infection of human B cells with LMP2A KO viruses led to a marked decrease in activation and proliferation, as well as higher levels of apoptosis, which led to inefficient long-term growth of the infected B cells in culture. LMP2B did not play a significant role in B cell activation, proliferation, or survival in early infection, nor was it necessary for long-term growth of infected B cells. The loss of LMP2A and LMP2B expression did not significantly affect latent gene expression, with the exception of LMP2B transcript in Δ2A-infected cells, nor did these genes appear to regulate latency and lytic induction in early infection. Our results suggest that LMP2A augments activation, proliferation and survival of B cells following EBV infection, which affects the ability of EBV to provide infected cells with an environment conducive to long-term outgrowth. In contrast, LMP2B does not significantly affect B cell activation, nor does this protein play a major role in proliferation and survival during early infection, as long-term outgrowth occurs similarly to wt.

## Materials and Methods

### Generation of Recombinant EBV viruses

Recombinant wild-type (wt) EBV (p2089) [50] and LMP2A knockout (KO) BACs (p2525) [35] were a generous gift from Wolfgang Hammschmidt and Markus Altmann (Department of Gene Vectors, Helmholtz Center, Munich, Germany). EGFP and hygromycin resistance genes were inserted into the recombinant wt and LMP2 KO EBV BACs for tracking infectivity of virus stocks and selection, respectively [50]. Flag tags were inserted into p2089 (wt) and p2525 (Δ2A) for these studies using a technique from W. Hammerschmidt [described in [51]]. Specifically, 10kb fragment of EBV sequence that corresponded to the LMP2 region was cloned into the shuttle vector p2768.5 (produced by M. Altmann and W. Hammerschmidt). This vector was used for insertion of a 3X flag tag into exon 7 and a zeocin resistance gene between exons 8 and 9 of the LMP2 gene. Homologous recombination between EBV sequence in p2768.5 and p2089 or p2525 created flag-tagged constructs p2089.1 (wt) and p2525.1 (Δ2A). For removal of LMP2B, the 10kb EBV DNA fragment in p2768.5 was constructed with LoxP sequences flanking exon 1′ of LMP2B. Exon 1′ was removed via recombination between flanking LoxP sequences after introduction of Cre recombinase; the new constructs were named p2089.3 (Δ2B) and p2525.3 (Δ2A/Δ2B).

### LMP2A and LMP2B Mutation Validation using PCR

The integrity of the LMP2A and LMP2B deletion mutations were assessed using qcPCR. First, the wt and LMP2 KO BACs were dialyzed in dH_2_O to remove TE storage buffer. Primers specific to the LMP2A and LMP2B exon 1 regions were used to determine the presence or absence of the respective first exons (Table 1). Standard PCR conditions and ABI GeneAmp PCR System 9700 instrument were used.

**Table 1:** Primers for Characterization of LMP2 knockout BACs.

Primer Name	Forward Primer (5′-3′)	Reverse Primer (5′-3′)
**2A Exon 1 Deletion**	ATC CCT CTC GCC TTG TTT CTC	GAT GGT GTG GAT AAC ATC TCC
**2B Exon 1 Deletion**	GCG GTG TGT GTG TGC ATG TAA GCG T	ACC TCA TTC TGA AAT TCC CAT ATC C

### Cell Lines, Cell Culture, and Reagents

HEK 293 cell line (generous gift from Robert White and Martin Allday, Imperial College London, UK, described in [52]) used for production of recombinant wt and LMP2 KO EBV BAC viruses as described previously [12]. All HEK 293 cells were cultured in D10 (DMEM, 10% FBS, 2 mM L-glutamine, 100 units/ml penicillin, 100 ug/ml streptomycin). HEK 293 cells that contain EBV BAC were cultured in D10+75 ug/ml hygromycin. PBMCs were purified from buffy coats of healthy, random donors by Ficoll-Hypaque density-gradient centrifugation. B cells were purified from PBMC using negative selection (B cell Isolation Kit II, Miltenyi Biotec), and cultured in R10 (RPMI-1640, 10% FBS, 2 mM L-glutamine, 100 units/ml penicillin, 100 ug/ml streptomycin). BCR ligation performed using 10 μg/ml anti-human IgG/A/M antibodies (Jackson Immunoresearch Laboratories) (as described in [53], [54].

### EBV Production and Titering

Recombinant wt and LMP2 KO EBV BACs (6 ug of each) were transfected into HEK 293 cells using Genejuice (Novagen). After 48hrs, EGFP-expressing HEK 293 were selected using hygromycin at 75 ug/ml. Lytic EBV production was induced by introduction of BZLF1 (Zebra) [24] and BALF4 (gB) [55] expression plasmids via transfection using Genejuice reagent (Novagen). Following 5–6 day incubation, supernatants were harvested, filtered through 0.45 μm filters, and titered by EGFP expression using Raji B cells as described previously [12], [55]. For EBV genome copy number, virions were lysed with 10 mM tris (pH 7.6) containing 50 mM KCl, 2.5 mM MgCl_2_, 1% Tween 20 and 0.1 mg/ml proteinase K. The lysed supernatants were used to detect a sequence in the BLLF1 gene that encodes the major viral glycoprotein gp350. The primers used for Real Time PCR were as follows: forward primer 5′-GTATCCACCGCGGATGTCA-3′; reverse primer 5′-GGCCTTACTTTCTGTGCCGTT-3′; and probe 5′-FAM-TGGACTTGGTGTCACCGGTGATGC-TAMRA-3′ [56]. Standards were used as mentioned previously [56]. Real time PCR performed with ABI 7500 instrument. All supernatants used in these studies were characterized and are presented in Table 2.

**Table 2:** Characterization of recombinant wt and LMP2 knockout EBV virus stocks.

EBV	Genome Copy #/ml	*GIU/ml	Genome Copy #/GIU
**wt**	4.3×10^7^	1.9×10^6^	23
**wt**	1.01×10^8^	4.1×10^6^	24.6
**Δ2B**	6.1×10^7^	4.8×10^6^	13
**Δ2B**	10×10^6^	5.45×10^5^	18.4
**Δ2B**	15.1×10^6^	5.6×10^5^	27
**Δ2B**	7.8×10^7^	2×10^6^	39
**Δ2A**	6.2×10^6^	1×10^6^	6.2
**Δ2A**	5.1×10^7^	9.5×10^5^	53.7
**Δ2A**	1.14×10^8^	2×10^6^	56.9
**Δ2A/Δ2B**	1.3×10^6^	8.8×10^5^	1.5
**Δ2A/Δ2B**	2.3×10^8^	2×10^6^	115
**Δ2A/Δ2B**	1.13×10^6^	7.9×10^5^	1.4
**Δ2A/Δ2B**	12.1×10^6^	6×10^6^	20.2

* GIU = Green Inducing Units, a measure of infectivity for the virus stocks.

### Real Time RT-PCR for EBV Gene Expression

Latent gene expression in HEK 293-EBV cell lines was confirmed using real time PCR. 1×10^6^ HEK 293-EBV cells were harvested for RNA extraction (Qiagen RNeasy Mini Kit) and cDNA conversion (High Capacity cDNA Kit, ABI). Real time PCR targets included EBNA1, EBNA2, EBNA3A, EBNA3B, EBNA3C, LMP1, LMP2A, and LMP2B. Each target transcript was normalized to cell number by measuring β_2_-microglobulin cDNA (primer set from Applied Biosystems) (described previously in [56]). All primer and probe sequences are supplied in Table 3. PCR for HEK 293 cell lines was performed using an ABI 7500 instrument. To assess kinetics of early EBV gene expression for recombinant wt and LMP2 KO EBV in infected B cells, 1×10^6^ B cells were infected at a multiplicity of infection (MOI) of 1. 100,000 infected B cells were harvested at 12, 24, 48, 72, 96, 120 and 168 hours post-infection, for RNA extraction (Qiagen RNeasy Mini kit) and conversion to cDNA (High Capacity cDNA kit, ABI). The EBV targets for infected B cells included EBNA1, EBNA2, LMP1, LMP2A, LMP2B and Zebra. Primers and probes are described in Table 3. PCR for EBV-infected B cells was performed using PerfeCTa® qPCR FastMix® II, Low ROX™ (Quanta Biosciences) per manufacturer instructions with an ABI ViiA 7 instrument. β_2_-microglobulin was used as a housekeeping gene for normalization of EBV gene mRNA in these experiments (described previously in [56]). Infection efficiencies for wt and LMP2 KO viruses were determined by EGFP expression, and were used as a correction factor for calculation of mRNA copies per infected cell.

**Table 3:** Primers and Probes for Real Time PCR analysis of EBV gene expression.

EBV Gene	Primer Sequence (5′-3′)	*Probe Sequence (5′-3′)
**EBNA1**	GAT TCT GCA GCC CAG AGA GTA GTC	TCG TCG CAT CAT AGA CCG CCA GTA GAC
	TCG TCA GAC ATG ATT CAC ACT TAA AG	
**EBNA2**	TAA CCA CCC AGC GCC AAT C	CAC CAC GTC ACA CGC CAG TGC TGG GT
	GTA GGC ATG ATG GCG GCA G	
**EBNA3A**	GAT TCT GCA GCC CAG AGA GTA GTC	CCC GGC CTG TCC TTG TCC ATT TTG
	CTT CTT CCA TGT TGT CAT CCA GG	
**EBNA3B**	GAT TCT GCA GCC CAG AGA GTA GTC	TAG ACC GCC AGT AGA CCT GGG AGC AGA
	CCA CGC TTT CTT CAT TAT TCA GGT	
**EBNA3C**	GAT TCT GCA GCC CAG AGA GTA GTC	AAG ACC CAC CAT GGA ATC ATT TGA AGG A
	CCA GGG TCC TGA TCA TGC TC	
**LMP1**	TCA TCG CTC TCT GGA ATT TG	AGC ACA ATT CCA AGG AAC AAT GCC TGT C
	TCC AGA TAC CTA AGA CAA GTA AGC AC	
**LMP2A**	CTA CTC TCC ACG GGA TGA CTC AT	TGT TGC GCC CTA CCT CTT TTG GCT GGC G
	GGC GGT CAC AAC GGT ACT AAC T	
**LMP2B**	CGG GAG GCC GTG CTT TAG	TGT TGC GCC CTA CCT CTT TTG GCT GGC G
	GGC GGT CAC AAC GGT ACT AAC T	
**BZLF1**	TTC CAC AGC CTG CAC CAG T	CAA CAG CCA GAA TCG CTG GAG GAA TGC G
	AGC AGC CAC CTC ACG GTA GT	

* All probes were conjugated with 5′ FAM and a 3′ TAMRA quencher.

### Flow Cytometry for Early Infection Kinetics

For all kinetics experiments, purified B cells were infected with wt and LMP2 KO EBV at MOI 1. To quantify proliferation kinetics, B cells were labeled with a proliferation dye (Vybrant DiI, Invitrogen) and harvested for analysis at 4, 8 and 16 days post-infection, and fixed with 1% paraformaldehyde. For activation kinetics studies, B cells were stained with anti-CD23-AlexaFluor647 and anti-CD71-APC antibodies at 0, 4, 8 and 16 days post-infection. For cell counts and apoptosis studies, B cells were stained with Violet Tracer (Invitrogen) at concentration of 2.5 μM, harvested at 4, 7 and 14 days post-infection and stained with Aqua Live/Dead cell indicator (Invitrogen) and AnnexinV-APC (Invitrogen) for apoptosis, which was performed according to the manufacturer's protocol. B cell samples were then fixed with 1% paraformaldehyde and prior to acquisition, a fixed number of cell counting beads (10 μl) were added (CountBright Absolute Counting Beads, Invitrogen) to obtain absolute count of proliferating B cells in each sample with normalized volume. Accordingly, acquisition was stopped at a collection of 5000 beads. All samples were acquired by FACSAria or FACSLSRII (BD Biosciences), and data were analyzed by FACSDiva and FlowJo 7.6.1 software.

### Long-term Outgrowth Assay

To determine the effect of LMP2 on the long-term outgrowth of infected B cells, 5×10^4^ negatively enriched B cells were infected with wt and LMP2 KO viruses at MOI of 1. Infected B cells were observed regularly over the course of 12–14 weeks to determine if efficient proliferation and LCL formation occurred. Standard proliferation assays are followed for approximately 6–8 weeks, but slow proliferation rates of LMP2A KO infected B cells required longer observation. At the end of 12–14 weeks, the number of wells with proliferating cells were determined, counted and transferred to 25 cm^2^ flasks.

## Results

### Construction and characterization of recombinant LMP2 KO EBV

**Figure 1:**
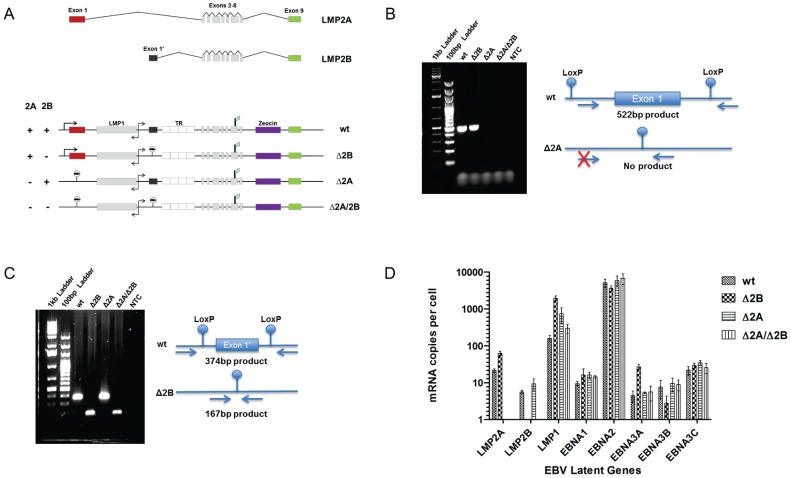
Recombinant BACs have deleted LMP2 regions and retain latent gene expression. (A) LMP2 knockout EBV BACs were created by deletion of LMP2A and LMP2B transcription initiating exons (Exon 1 and Exon 1′). LoxP sites were inserted, flanking each initial exon, and recombined via introduction of Cre Recombinase expression plasmid (pKD46) into DH10B bacteria containing floxed BACs. (B) Recombinant wt and Δ2B BACs contain LMP2A Exon 1, which is demonstrated by presence of 522 bp PCR band. Δ2A and Δ2A/Δ2B BACs have deleted Exon 1, which is demonstrated by absence of PCR band due to removal of the forward primer-binding site. (C) Recombinant wt and Δ2A BACs contain Exon 1′, which is demonstrated by 374 bp PCR band. Δ2B and Δ2A/Δ2B BACs have deleted Exon 1′, evidenced by the presence of 167 bp PCR deletion band. (D) Recombinant wt and LMP2 knockout EBV BACs transfected into HEK 293 cells. After establishment of BAC-containing cell lines, each cell line was assessed for latent gene expression using Real Time PCR. Δ2B cell lines were negative for LMP2B transcript, Δ2A cell lines were negative for LMP2A transcript, and Δ2A/Δ2B cell lines were negative for both transcripts. Expression of latent genes was similar for wt and LMP2 knockout BACs.

In order to investigate the contribution of both LMP2 isoforms to early events in EBV infection of primary B cells *in vitro*, LMP2A and LMP2B knockout viruses were generated. Δ2A (p2525) EBV BAC was constructed by the Hammerschmidt group [35] (Figure 1A). Δ2B BACs were constructed using either the wt or Δ2A BACs as backbones for insertion of LoxP sequences directly upstream and downstream of exon 1′ of the LMP2B gene. The introduction of Cre recombinase resulted in recombination of the flanking LoxP sites and subsequent removal of the exon creating Δ2B and Δ2A/Δ2B (Figure 1A). The deletions of exon 1 (LMP2A) and exon 1′ (LMP2B) were confirmed by PCR (Figure 1B and 1C). Specifically, the LMP2A deletion was confirmed using a forward primer within the 5′ floxxed region that contains the LMP2A promoter and a reverse primer outside the 3′ floxxed region. In BACs that are wild type for the LMP2A gene, both forward and reverse primers yield a 522 bp PCR product. However, in Δ2A BACs, the internal floxxed region, which includes the binding site for the forward primer, has been removed and does not yield a PCR product. The LMP2B deletion was confirmed using forward and reverse primers outside the floxxed region that yielded 374 bp product in wt LMP2B BACs and a smaller 167 bp product in Δ2B BACs. Since transcription of LMP2A and LMP2B initiates in the deleted exon 1 and exon 1′ respectively, removal of those exons lead to a complete loss of LMP2A and LMP2B transcripts (Figure 1D).

The recombinant BACs were transfected into HEK 293 cells for virus production. After 1–2 months under hygromycin selection, the producer cell lines were screened for latent gene expression to assess the integrity of the LMP2 KO BACs. The mRNA expression levels of 5 EBNAs (EBNA1, EBNA2, EBNA3A, EBNA3B and EBNA3C) and 3 LMP genes (LMP1, LMP2A and LMP2B) were verified using quantitative real time PCR (Figure 1D). Target EBV genes were normalized to a cellular housekeeping gene, β_2_-microglobulin, and expressed as mRNA copies/cell, as noted previously [56]. The Δ2A and Δ2A/Δ2B producer cell lines were negative for LMP2A transcripts, while the Δ2B and Δ2A/Δ2B cell lines were negative for LMP2B transcripts (Figure 1D). These results demonstrate that the mutations in the BACs have the knockout phenotypes expected. The expression levels for the other latent genes were similar between all wt and LMP2 KO BAC-containing producer cell lines with only minor variations in EBNA3A and LMP1 gene expression (Figure 1D).

### Production of recombinant EBV and characterization of virus stocks

The producer cell lines were seeded for transfection of two expression plasmids to induce lytic reactivation. One expression plasmid produced the EBV lytic transactivator gene BZLF1, or Zebra, while the other produced one of the EBV glycoproteins called gB. Introduction of these two plasmids into the HEK 293-EBV producer cell lines induces lytic reactivation and production of mature, infectious virions [24], [55]. The supernatants containing the viruses were harvested after 5–6 days incubation, and infectious titer was determined by incubating Raji B cells with the virus supernatants [12], [55]. Characterization of the virus stocks included determining the genome copy number using Real Time PCR primers specific for the major virus glycoprotein gp350. This allowed us to selectively use recombinant virus stocks with similar genome copy number to infectious unit (copy#/GIU) ratios for subsequent experiments that reduced the potential for differences in the kinetics to be attributable to variation in particle doses (Table 2).

### Δ2A viruses do not induce efficient B cell proliferation in early EBV infection

**Figure 2:**
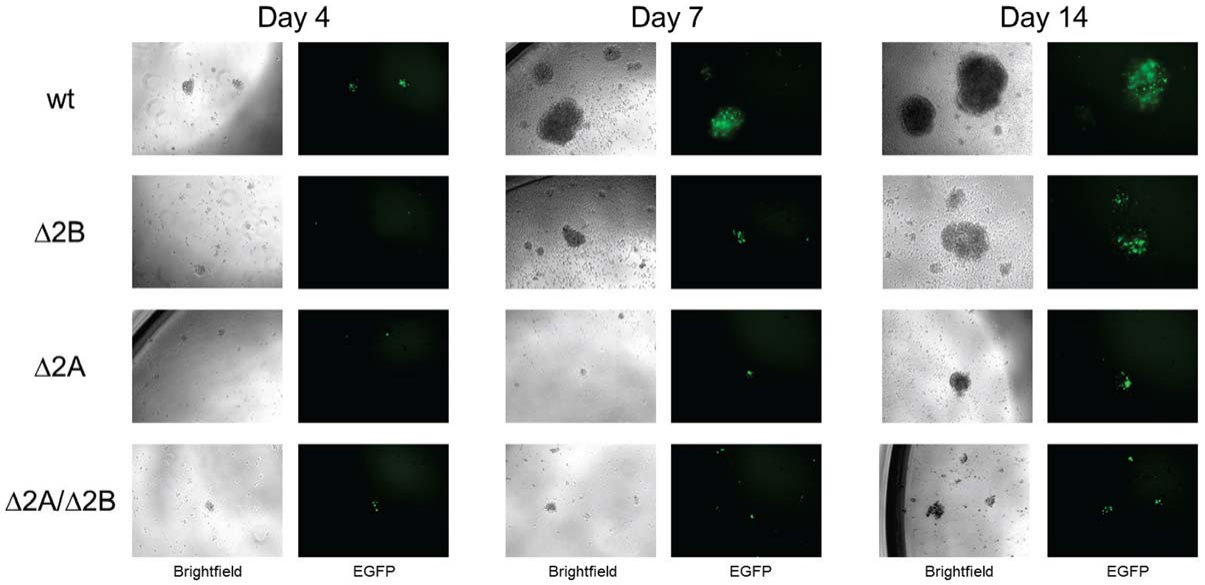
Proliferation of B cells infected with wt and LMP2 KO viruses. 5×10^4^ B cells infected with recombinant wt and LMP2 KO EBV at MOI 1. EGFP expression and proliferation was examined at 4, 7 and 14 days post-infection using inverted fluorescent microscope.

**Figure 3:**
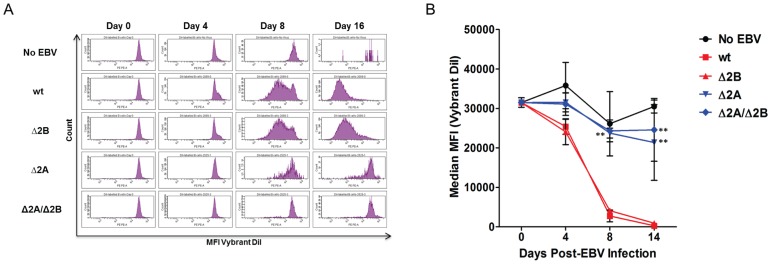
Quantitation of B cell proliferation following wt and LMP2 KO virus infection. To quantify B cell proliferation, 1×10^6^ B cells labeled with proliferation dye (Vybrant DiI, Invitrogen) were infected with recombinant wt and LMP2 KO EBV at MOI 1. (A) Infected B cells were harvested and analyzed at 4, 8 and 16 days post-infection using flow cytometry. (B) Each data point is an average of 3 independent experiments ± SEM. Statistical significance was determined using Two-way ANOVA and Bonferroni Post-test. P-value (**) <0.01 for both Δ2A and Δ2A/Δ2B viruses compared to wt at 8 and 16 days post-infection. No significant differences between proliferation of wt and Δ2B-infected B cells.

It has previously been shown that wt EBV efficiently induces proliferation of human B cells *in vitro* [1], [57]. In addition to the overall immortalizing capabilities of EBV, the early events associated with B cell infection by wt EBV have been elucidated [57]. Although the LMP2 gene is always expressed, it has not been clearly demonstrated what role LMP2A and LMP2B play in early B cell infection. In order to investigate the roles of LMP2A and LMP2B in this process, B cells were infected with recombinant wt and LMP2 KO viruses at an MOI of 1, and monitored for approximately 2 weeks for EGFP expression, cell activation, clumping and proliferation (Figure 2). By approximately 4 days post-infection, EGFP expression was observed in B cells at similar levels and green cells coalesced into small, scattered clumps (<10 cells) that were present in all virus treated wells. Over time, clumps increased in size in wells infected with wt and Δ2B viruses, but were larger and more numerous in wt-infected wells. There was a lack of significant increase in the size of clumps in Δ2A or Δ2A/Δ2B virus-treated wells during the observation period (Figure 2). DiI proliferation tracker experiments revealed that wt and Δ2B infected cells began to proliferate between 4 and 8 days post-infection (Figure 3A). By 8 and 16 days post-infection high levels of proliferation were observed in wt and Δ2B virus-infected B cells (Figure 3A). Repeat experiments conducted at different times using different B cell donors gave similar results (Figure 3B). The amount of proliferation detected in the cytometry experiments correlated with the appearance and size of clumps formed in undisturbed wells. However, Δ2B-infected wells seemed to lag slightly behind wt in both clump size (Figure 2) and mean fluorescence intensity (Figure 3A), but the effect was too small to be demonstrated with a statistical significance. Efficient proliferation was not observed in wells containing B cells infected with either of the LMP2A KO viruses (Δ2A p-value <0.01, Δ2A/Δ2B p-value <0.01) (Figure 3B). Therefore, our results suggest that the inclusion of LMP2A in our recombinant viruses is advantageous for efficient proliferation of infected B cells in early infection.

### Δ2A viruses do not induce efficient B cell activation

**Figure 4:**
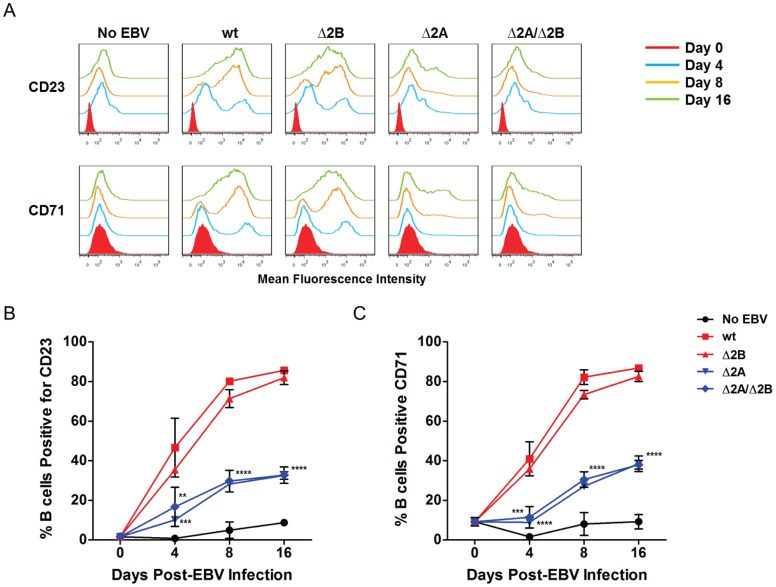
LMP2A is critical for sufficient activation of B cells. 1×10^6^ purified B cells infected with recombinant wt and LMP2 KO EBV at MOI 1. B cells harvested at 4, 8 and 16 days post-infection for analysis of activation marker expression. (A) Expression of CD23 (marker of B cell activation) and CD71 (marker of lymphocyte activation and proliferation) assessed by flow cytometry and analyzed using FlowJo 7.6.1 software. (B) Expression of CD23 and (C) CD71 quantified and expressed as percent positive for surface markers. Each data point is an average of 2 independent experiments ± SEM. Statistical significance was determined using Two-way ANOVA and Bonferroni Post-test (each data point for LMP2 KO viruses was compared to wild-type infection to determine statistical significance). P-value (**) <0.01 for Δ2A CD23 expression at 4 days post-infection. P-value (***) <0.001 for Δ2A/Δ2B CD23 expression at 4 days post-infection and Δ2A CD71 expression at 4 days post-infection. P-value (****) <0.0001 for Δ2A CD71 expression at 4 days post-infection, and Δ2A and Δ2A/Δ2B viruses for both CD23 and CD71 expression at 8 and 16 days post-infection.

Since there were clear differences in the proliferation kinetics associated with Δ2A and Δ2A/Δ2B viruses in early EBV infection, and to a lesser extent the Δ2B virus, the effect of LMP2A and LMP2B on B cell activation was examined. Previous studies have shown that LMP2A can produce a signal that mimics an activated BCR signal, which is demonstrated by its ability to activate PTKs and Ca^2+^ initiation complexes that result in Ca^2+^ fluxes resembling those observed after BCR stimulation [39]. Activation of uninfected B cells can be triggered by BCR stimulation following antigen recognition and a second signal, such as CD40-CD40L signaling [58]. Therefore, it is reasonable to hypothesize that LMP2A may play a role in B cell activation, and may act in concert with LMP1 to induce activation following *in vitro* infection. For this experiment, infected B cells were probed for expression of two surface markers specific for B cell activation and proliferation, CD23 and CD71, at 0, 4, 8 and 16 days post-infection. By 4 days post-infection, higher percentages of B cells infected with wt and Δ2B viruses had upregulated levels of CD23 and CD71, indicating efficient B cell activation (Figure 4A). The proportion of cells expressing CD23 and CD71 increased throughout the entire 16-day experiment, with levels reaching as high as 80–85% B cells positive for the two surface markers (Figure 4B and 4C). Initially, a smaller percentage of Δ2A and Δ2A/Δ2B virus-infected B cells showed CD23 and CD71 expression, which also displayed delayed expression kinetics that did not increase similarly to wt-infected B cells over time (Figure 4A). At 16 days post-infection only 30–40% of B cells were positive for the activation markers (CD23 Δ2A 4-day p-value <0.001, Δ2A 8-day and 16-day p-value <0.0001) (CD23 Δ2A/Δ2B 4-day p-value <0.01, Δ2A/Δ2B 8-day and 16-day p-value <0.0001) (CD71 Δ2A 4, 8, 16-day p-value <0.0001) (CD71 Δ2A/Δ2B 4-day p-value <0.001, Δ2A/Δ2B 8-day and 16-day p-value <0.0001) (Figure 4B and 4C). Therefore, we conclude that LMP2A is involved in the process of B cell activation following EBV infection.

### LMP2A KO virus infections exhibit higher percentages of apoptotic B cells

These observations suggested that there was a critical role for LMP2A in early activation and proliferation of primary, human B cells following EBV infection. Several studies have established a role for LMP2A in B cell survival [33], [34], [35]. The BCR-like signal that LMP2A provides infected B cells may mimic the effects of the tonic BCR signaling that is essential for B cell survival [34], [59]. Therefore, we hypothesized that the deficiencies in activation and proliferation of B cells by Δ2A and Δ2A/Δ2B viruses could be due to increased apoptosis as a consequence of defective B cell survival signals. To examine this, purified B cells were labeled with Violet Tracer proliferation dye (Invitrogen) and infected with recombinant wt and LMP2 KO viruses. A violet proliferation dye, in place of Vybrant DiI (Invitrogen), was used for these experiments due to its enhanced ability to trace multiple generations of proliferating cells and, therefore, produce more accurate measures of the numbers of proliferating cells over time. Analysis using median MFI for Vybrant DiI experiments showed slight differences in B cell proliferation kinetics between wt and Δ2B viruses, but did not reach statistical significance (Figure 3A and 3B). However, the differences in proliferating B cell numbers in Δ2B and wt virus wells were more evident in the size and number of clumps in live microscopy images (Figure 2). Cells were collected at 4, 7 and 14 days post-infection for analysis, and stained with Aqua live/dead cell indicator and Annexin V for apoptosis. The cells were incubated with a specific number of Absolute Cell Counting Beads (Invitrogen) to standardize the acquisition volume of the sample on the cytometer (FACSLSRII), which allowed for absolute cell counts of proliferating B cells.

**Figure 5:**
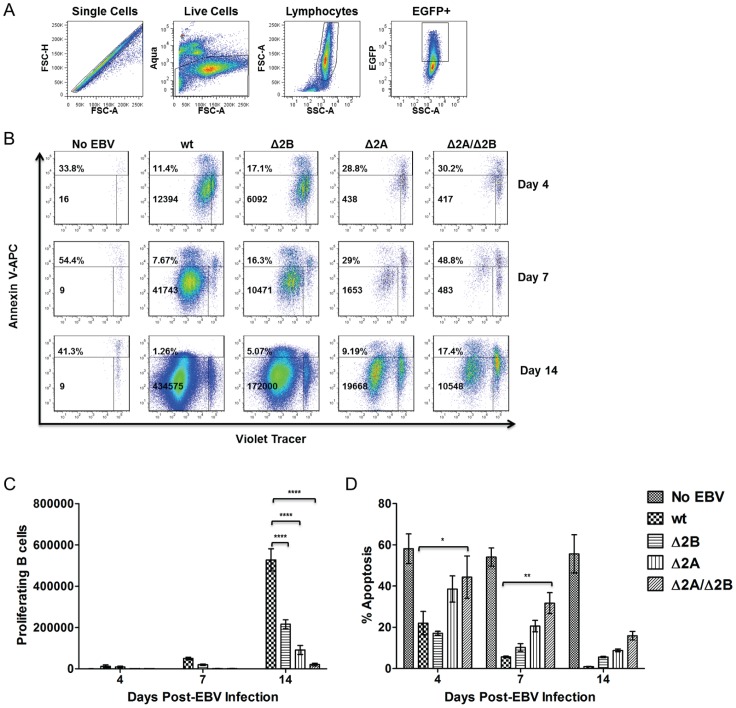
Efficient Proliferation and Survival of EBV-infected B cells require LMP2. 1×10^6^ purified B cells were labeled with Violet Tracer (Invitrogen) and infected with recombinant wt and LMP2 KO EBV at MOI 1. B cells were harvested at 4, 7 and 14 days post-infection and stained for Annexin V. During fixation step, 10 μl counting beads (CountBright Absolute Counting Beads, Invitrogen) were added to each sample. During acquisition, the event gate was set to 5000 beads, which normalized the acquisition volume between samples and allowed for accurate, absolute counts of proliferating B cells. (A) Gating strategy for proliferation and apoptosis analysis. Doublets and dead cells were excluded. Next, lymphocytes and EGFP+ cells were selected for proliferation and apoptosis analysis. (B) Representative donor for proliferation and apoptosis data. (C) Proliferation and (D) Apoptosis data points are an average of 3 independent experiments ± SEM. Statistical significance was determined using a Two-Way ANOVA test and Bonferroni Post-test (each data point for LMP2 KO viruses was compared to wild-type infection to determine statistical significance). P-value (*) <0.05, p-value (**) <0.01 and p-value (****) <0.0001.

In order to better quantify these differences using flow cytometry, we collected proliferation and apoptosis levels simultaneously for wt and LMP2 KO virus-infected cells from the same donor. For analysis, we used a series of gates to eliminate specific B cell populations (Figure 5A), such as doublet, necrotic and uninfected cells. With this gating strategy, the Violet Tracer and Annexin V gates were specific for the proliferating and apoptotic EBV-infected cells within the B cell population. Using these selection gates, results from three experiments (and three different donors) were pooled. B cells infected with wt virus exhibited the highest levels of proliferation. The absolute number of proliferating wt-infected B cells was ≥2x the number of proliferating B cells in Δ2B wells (p-value <0.0001), and ≥5× and ≥10× the number of proliferating B cells in Δ2A (p-value <0.0001) and Δ2A/Δ2B (p-value <0.0001) wells, respectively (Figure 5B and 5C). Measuring absolute numbers of proliferating B cells demonstrated a significant difference in LMP2 KO virus-infected proliferating B cells compared to wt-infected cells. We next compared these proliferation results to the rates of apoptosis detected in the three separate trials. Wt-infected B cells exhibited the lowest percentage of apoptotic B cells (Figure 5B and 5D), Δ2B virus-infected B cells had slightly higher levels of apoptosis compared to wt-infected B cells (Figure 5B and 5D) and Δ2A virus-infected B cells consistently had much higher levels of apoptosis compared to both wt and Δ2B viruses (Figure 5B and 5D). Deletion of both LMP2 isoforms resulted in the highest levels of apoptosis (Day 4 p-value <0.05, Day 7 p-value <0.01) and lowest levels of proliferation (Figure 5C and 5D). Together, the data indicate that there is an inverse relationship between the amount of apoptosis and proliferation occurring in the virus-infected populations, and suggest that LMP2A promotes the survival of EBV-infected B cells, particularly within the first week of infection. LMP2B alone did not significantly affect B cell survival, but removal of both protein isoforms appears to increase the percentage of apoptotic B cells, an effect that was also most readily observed within the first week of infection.

### LMP2 KO viruses do not alter EBV gene expression profiles

**Figure 6:**
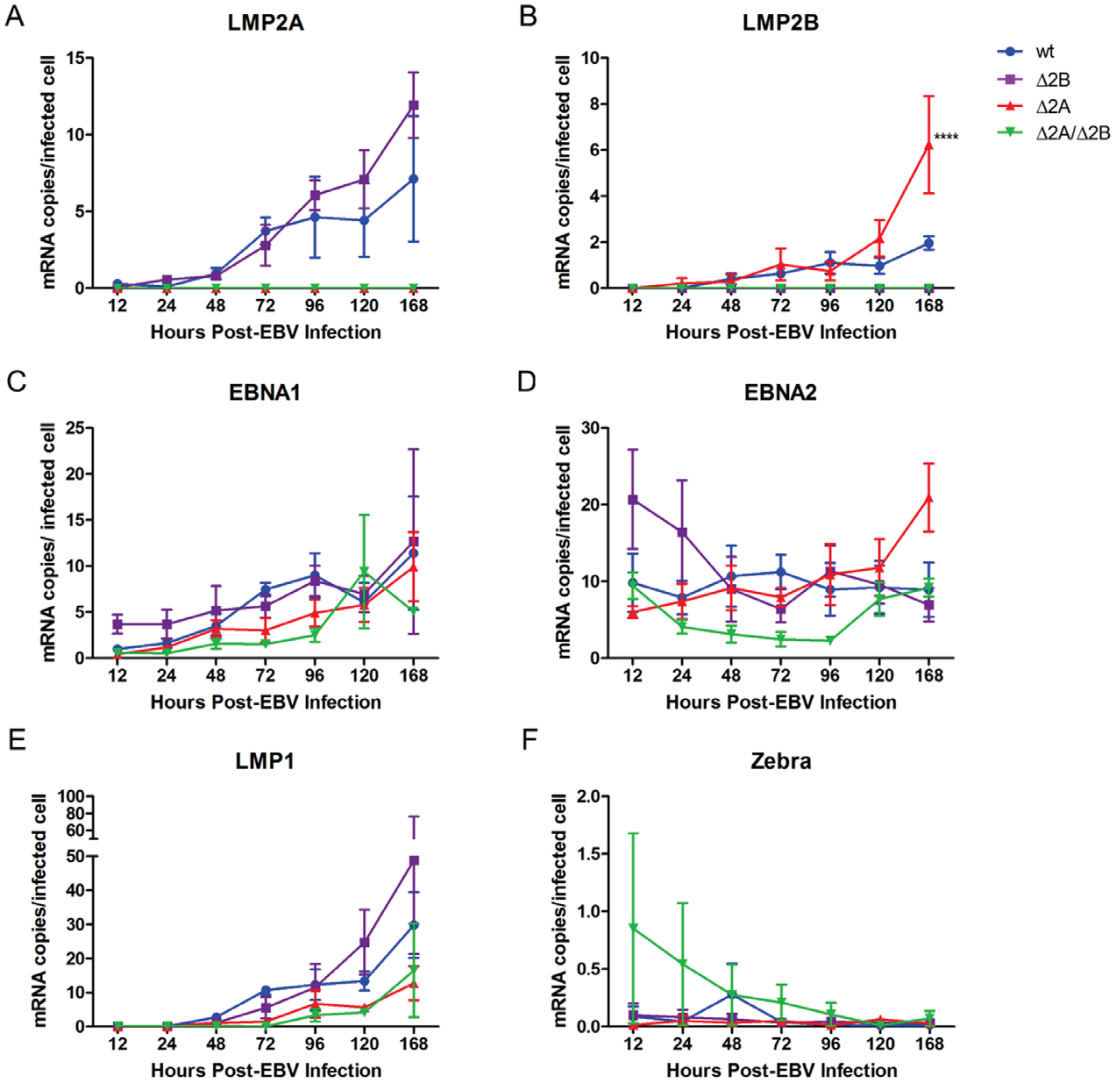
EBV Gene Expression in early EBV infection with LMP2 KO viruses. 1×10^6^ purified B cells were infected with recombinant wt and LMP2 KO EBV at MOI 1. (A) RNA was harvested for analysis at 12, 24, 48, 72, 96, 120 and 168 hours post-infection and analyzed by Real Time PCR to determine the effect of LMP2 on latent gene expression. Specifically, we examined the latent genes LMP2A, LMP2B (B), EBNA1 (C), EBNA2 (D), LMP1 (E) for changes in early gene expression. (F) In addition to latent genes, the lytic gene BZLF1 or Zebra, an immediate early lytic transactivation gene, was examined. β_2_Microglobulin mRNA was amplified for EBV gene normalization. Each data point is an average of three independent experiments ± SEM. Statistical significance determined using Two-way ANOVA and Bonferroni Post-test (each data point for LMP2 knockout viruses was compared to wild-type infection to determine statistical significance). P-value (****) <0.0001.

To further explore the effect of LMP2 KO viruses on early EBV infection, we hypothesized that the effects observed in activation, proliferation, and survival experiments could be due to altered expression of other EBV latent and lytic genes involved in proliferation and/or survival following deletion of LMP2. For instance, previous studies suggested a possible regulatory role for LMP2A in EBV latent gene expression as a way of evading T cell recognition of latent gene epitopes [60], and LMP2B has been shown to have regulatory effects on lytic induction [48], [49]. Previous studies have shown that LMP2A expression coincides with stability in viral latency, therefore, we also hypothesized that loss of LMP2 could lead to instability of viral latency and subsequent loss of proliferation. Therefore, B cells were infected with recombinant wt and LMP2 KO EBV at MOI of 1 and were harvested at 12, 24, 48, 72, 96, 120 and 168 hours post-infection to assess differences in gene expression for the latent genes EBNA1, EBNA2, LMP1, LMP2A, LMP2B and the lytic transactivator, Zebra. As expected, Δ2A and Δ2A/Δ2B virus-infected B cells were negative for LMP2A expression (Figure 6A), and Δ2B and Δ2A/Δ2B virus-infected B cells were negative for LMP2B expression (Figure 6B). Wt and Δ2B viruses displayed similar levels of LMP2A expression over the course of the experiment (Figure 6A). Δ2A-infected B cells also had similar levels of LMP2B expression at early time points compared to wt, but demonstrated significantly higher expression at the latest time point (168 hour p-value <0.0001) (Figure 6B).

Wt and Δ2B virus-infected B cells exhibited similar expression levels of EBNA1 (Figure 6C), EBNA2 (Figure 6D) and LMP1 (Figure 6E) over the 1-week experimental period. The observed trends in early gene expression correlated with previous published data for these genes in wt EBV infection [57]. Expression of EBNA1 gradually increased over the course of the week while LMP1 was low at early time points and increased over the 1-week period. EBNA2 expression was slightly higher at early time points and gradually stabilized over time. The LMP1 expression data demonstrate that the deletion of LMP2B exon 1′ did not negatively affect regulation of the bidirectional promoter that is shared with LMP1. EBNA1 and EBNA2 expression in Δ2A and Δ2A/Δ2B-infected B cells were consistently lower, albeit not statistically significant, compared to wt at early times but became comparable to wt near the end of the observation period (Figure 6C, 6D). The levels of LMP1 expression were initially lower for Δ2A and Δ2A/Δ2B viruses, though not statistically significant, compared to wt, but increased throughout the experiment with kinetics similar to wt and Δ2B (Figure 6E). Therefore, these data suggest that the removal of LMP2A and LMP2B does not affect expression of other EBV latent genes, except for an increase in LMP2B transcription in the absence of LMP2A.

In addition to latent gene expression, we investigated the effect of LMP2 KO viruses on the maintenance of viral latency by measuring Zebra expression. Overall, Zebra levels were less than one copy per cell for all virus-infected B cell populations (∼0.1 copies/cell in wt, Δ2B and Δ2A infections, ∼0.1–0.8 copies/cell for Δ2A/Δ2B infections) (Figure 6F), indicating that removal of LMP2A and/or LMP2B did not alter the ability of EBV to establish viral latency within the infected B cell population during natural progression of infection *in vitro*.

### BCR stimulation affects ΔLMP2A and ΔLMP2B virus-infected B cells

**Figure 7:**
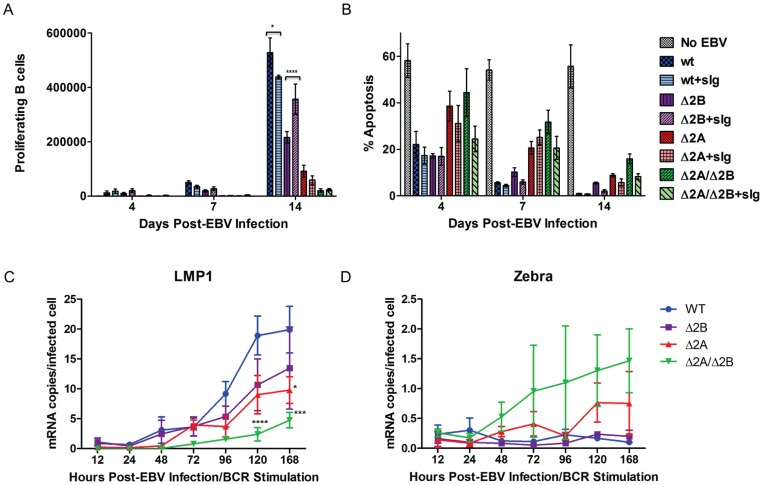
Effect of BCR stimulation on B cell infection with LMP2 KOs. 1×10^6^ purified B cells were labeled with Violet Tracer (Invitrogen) and infected with recombinant wt and LMP2 KO EBV at MOI 1. 10 ug/ml sIg (Jackson Immunoresearch) was added to each sample for BCR stimulation. B cells were harvested at 4, 7 and 14 days post-infection and stained for Annexin V. During fixation step, 10 μl counting beads (CountBright Absolute Counting Beads, Invitrogen) were added to each sample. During acquisition, the event gate was set to 5000 beads, which normalized the acquisition volume between samples and allowed for accurate, absolute counts of proliferating B cells. (A) Proliferation and (B) apoptosis data points are an average of three independent experiments ± SEM. (C) (D) RNA harvested at specific time points (12, 24, 48,72, 96, 120 and 168 hours post-infection/BCR stimulation) for analysis using Real Time PCR. (C) One latent gene (LMP1) and (D) one lytic gene (Zebra) analyzed for each time point to determine lytic induction. Statistical significance determined using Two-way ANOVA and Bonferroni Post-test (Figure 7C & 7D – each data point for LMP2 knockout viruses was compared to wild-type infection to determine statistical significance). P-value (*) <0.05, p-value (***) <0.001 and p-value (****) <0.0001.

The low spontaneous Zebra expression levels suggested that very few virus-infected cells in any of the infected populations were undergoing lytic cycle induction, which is consistent with previous studies that showed transient Zebra expression in early EBV infection did not induce the lytic cycle [61], [62]. However, it remained possible that the sensitivity to a lytic inducing signal was altered in LMP2 KO viruses. Previous studies have determined that stimulation of the BCR induces signaling and subsequent Ca^2+^ fluxes that trigger the EBV lytic cycle [63]. Therefore, we investigated the effect of BCR stimulation on proliferation, apoptosis and EBV lytic induction in B cells infected with wt and LMP2 KO viruses. In order to stimulate signaling through the BCR, infected cells were treated with 10 μg/ml soluble immunoglobulin (sIg). The infected/stimulated B cells were harvested at 4, 7 and 14 days post-stimulation for analysis using FACSLSRII as described above (Figure 5). Addition of sIg to wt-infected B cells had a small but significant effect reducing the number of proliferating B cells (p-value <0.05) (Figure 7A), while the number of apoptotic B cells was not significantly affected (Figure 7B). BCR stimulation induced significantly higher numbers of proliferating B cells in Δ2B virus-infected B cells (p-value <0.0001) (Figure 7A), but did not induce significant changes in apoptosis (Figure 7B). Δ2B virus-infected B cells demonstrated stable latent gene expression (Figure 7C) and low levels of lytic gene expression (Figure 7D), all of which were comparable to wt. BCR stimulation of LMP2A KO virus-infected B cells (Δ2A and Δ2A/Δ2B) did not significantly decrease the number of proliferating B cells (Figure 7A) or apoptotic B cells (Figure 7B). In contrast to wt and Δ2B viruses, BCR stimulation of Δ2A and Δ2A/Δ2B virus-infected B cells resulted in significantly reduced LMP1 expression when compared to wt-infected B cells (Δ2A 168 hours p-value <0.05, Δ2A/Δ2B 120 hours p-value <0.0001, Δ2A/Δ2B 168 hours p-value <0.001) (Figure 7C), though the observed effect was most significant for Δ2A/Δ2B infection. However, BCR stimulation of these cells did not significantly increase Zebra expression (Figure 7D). Therefore, removal of either LMP2A or LMP2B alone does not appear to significantly affect the ability of EBV to maintain latency following stimulation of BCR signaling. However, removal of both proteins significantly reduced expression of LMP1, possibly indicating instability in proliferative capacity.

### Removal of LMP2A affects long-term outgrowth of EBV-infected B cells

We reasoned that the effects observed for activation, proliferation and survival during the first two weeks following EBV infection could affect the long-term growth of infected B cells, a hallmark of wt EBV infection. Therefore, we infected purified B cells from six healthy donors with recombinant wt and LMP2 KO EBV at an MOI of 1, and observed proliferation for 12–14 weeks to account for slow proliferation kinetics for LMP2A KO viruses (Table 4). Wt-infected B cells established LCLs in approximately 5 weeks for all six donors. Within the context of our experimental system, we determined an LCL was ‘established’ when accumulation of proliferating B cells reached a point that required transfer into 25 cm^2^ flasks. Similar to wt EBV, Δ2B virus-infected B cells produced LCLs for all six donors within a 5–6 week period. In contrast to wt and Δ2B virus-infected B cells, B cells infected with Δ2A virus only established LCLs for three of the six donors. Additionally, establishing LCLs from two of the donors required approximately 12–14 weeks following infection due to slow rates of accumulation of proliferating infected B cells. Only B cells from one of the six donors infected with Δ2A/Δ2B viruses developed into LCLs, which also demonstrated slow accumulation rates that required 12–14 weeks before establishment. Therefore, our observations suggest that LMP2A is critical for efficient establishment of LCLs *in vitro*. Although LMP2B does not appear critical for establishment of LCLs, the loss of both proteins creates a phenotype that is the least conducive to formation of LCLs *in vitro*.

**Table 4:** Outgrowth Assay of Wt and LMP2 knockout EBV-infected B cells.

	Wt EBV	Δ2B EBV	Δ2A EBV	Δ2A/Δ2B EBV
**Donor 1**	+	+	+*	+*
**Donor 2**	+	+	+*	−
**Donor 3**	+	+	−	−
**Donor 4**	+	+	−	−
**Donor 5**	+	+	+	−
**Donor 6**	+	+	−	−

(+) Indicates establishment of LCLs within 5–6 weeks post-infection.

(−) Indicates LCLs were not established during the entire length of the experiment, which was equivalent to 12–14 weeks.

(+*) Indicates LCLs were established, but the length of time required was the total length of the experiment, equivalent to 12–14 weeks.

## Discussion

In this study, we have demonstrated that expression of LMP2A augments early activation and proliferation of EBV-infected B cells. This appears to be critical for subsequent establishment of LCLs, since loss of LMP2A expression correlates with reduced LCL formation (Table 4). Previous studies demonstrated the dispensability of LMP2A for establishment of LCLs [40], [41], [42], but did not include analyses of activation or proliferation in infected B cells during the early stages of infection. It is important to note that many of these studies employed heterogeneous populations of wild-type and LMP2 mutant EBV, which precluded clear assessments of the effects of LMP2 on B cell activation and proliferation from the time of initial infection. Other studies have used mini-EBV plasmids, which are incomplete EBV genomes that express all latent genes necessary for immortalization, and have shown that LMP2 was necessary for efficient immortalization of B cells [43]. Therefore our approach, which utilizes the complete EBV genome with deletions in the initiating exons of both LMP2A and LMP2B, provides a more comprehensive system in which to investigate the roles of the LMP2 isoforms in the activation and proliferation events that occur shortly after B cell infection that, ultimately, lead to establishment of continuously proliferating lymphoblastoid cell lines.

Previous studies have shown LMP2A delivers a signal that is suggested to mimic tonic BCR signaling [30], [34], [59], [64], [65] through the Ras/PI3-K/Akt pathway [65], which results in inhibition of apoptosis in infected B cells via induction of anti-apoptotic genes Bcl-2 and Bcl-xL [66], [67], [68]. Our data support a role for LMP2A in B cell survival that appeared to be important at the earliest times (4 to 7 days) after EBV infection when proliferation was initiating in the majority of infected cells. A role in survival seemed less critical by 14 days post-infection, which was suggested by the overall decrease in apoptosis percentages (Figure 5D). LMP2A may also mimic an activated BCR signal, inducing B cell activation and proliferation via Ca^+2^ fluxes and protein tyrosine kinase activation [39]. Both EBV-infected and BCR-activated cells express the surface activation markers CD23, CD40, CD44 and CD69 [69], [70], [71], [72], [73]. Therefore, it is possible that the signal provided by LMP2A cooperates with LMP1 and EBNA2 for the most efficient activation of B cells [69].

Efficient activation of primary B cells should lead to optimal early proliferation (within 4–5 days), which would be consistent with our data (Δ2A vs wt infection) and represent a critical factor for long-term LCL growth establishment *in vitro*. However, an exceedingly higher level of early cell proliferation (hyperproliferation) has been shown to suppress growth of wild-type EBV-infected primary B cells by activation of ATM kinase through induction of the DNA Damage Response (DDR), and it appears that the attenuation of DDR in a small percentage of EBV-infected cells allows for subsequent outgrowth and establishment of LCLs [62]. Interestingly, the Δ2A-infected B cells underwent early moderate proliferation, not hyperproliferation, compared to wt and Δ2B virues, and did not produce long-term LCLs as consistently. This suggests that high levels of early proliferation favor efficient LCL outgrowth. Overall, it raises the possibility that LMP2A is able to both trigger early attenuation of DDR while supporting a signaling environment that enhances robust early proliferation of infected B cells. Although the major factor in DDR attenuation is EBNA3C [62], a further investigation of a precise role of LMP2A in attenuation of DDR as well as enhancement of proliferation in early EBV-infected B cells is warranted.

The LMP2B isoform did not appear to be critical for B cell activation and proliferation since Δ2B-infected B cells exhibited a near wt level of activation and proliferation. This implies that most of the LMP2 gene effects on these processes are due to the signaling domain in LMP2A. There are possible mechanisms by which LMP2B may have effects on BCR or BCR-like signaling during infection. For instance, there are several lines of evidence showing that LMP2B can interact with signaling proteins such as CD19 [45], a member of the BCR co-receptor complex. In our experiments, LMP2B did not appear to play a direct role in events leading to activation, proliferation or protection from apoptosis of infected B cells. Although loss of both isoforms resulted in the highest levels of apoptosis, suggesting a possible role for LMP2B in survival of infected B cells, the exact role, if any, of this protein in early B cell infection remains unclear.

Previous research has demonstrated a role for LMP2A in the maintenance of viral latency [36], [64], [74]. Therefore, we reasoned that stimulation of BCR signaling in LMP2A KO virus-infected B cells would result in enhanced induction of the lytic cycle, which could partially explain the loss of efficient activation and proliferation of infected B cells. However, in the context of our experimental system, stimulation of BCR signaling in Δ2A-infected B cells did not result in an enhanced lytic switch, suggesting that LMP2A did not play an important role in maintenance of viral latency in the early stages of outgrowth *in vitro*. Based on previous research demonstrating that LMP2B regulated the lytic switch [48], [49], we had expected BCR stimulation of Δ2B-infected B cells to be more resistant to lytic reactivation. However, the observed levels of LMP1 and Zebra expression were comparable to wt, which suggests that Δ2B-infected B cells were not more resistant to lytic reactivation than wt. The loss of both isoforms, on the other hand, consistently triggered elevated Zebra expression with concomitant significant decreases in LMP1 expression, a protein that is essential for immortalization *in vitro* [12], [13]. Since this was not observed in B cells infected with either Δ2A or Δ2B viruses, it is possible that either LMP2A or LMP2B may be able to provide the maintenance function for viral latency *in vitro*. It is possible that strong lytic induction was not observed due to weak stimulation of BCR signaling. The use of chemical lytic inducers, such as histone deacetylase inhibitors or protein kinase C activators, could induce a stronger lytic signal that may allow for a better understanding of the role of LMP2A and LMP2B in the maintenance of viral latency in early B cell infections with recombinant virus.

The lack of significant differences in the expression of other EBV latent genes between wt and LMP2 KO virus-infected cells indicates that the recombinant viruses were subject to the same regulatory controls governing the expression of these genes as wt virus, and was another illustration of the stability of viral latency in these cells. Only LMP2B transcript levels significantly differed in Δ2A-infected B cells compared to wild-type. The increased LMP2B expression observed may imply the existence of an as yet unknown indirect regulatory mechanism requiring LMP2A. It is as likely that the effect is an artifact of the release of the LMP2B promoter from transcriptional repression caused by the drop in through-transcription from the upstream-mutated LMP2A promoter that allows improved RNA polymerase initiation. Heterogeneity within the B cell population and among donors could contribute to slight differences in gene expression, as well as differences in proliferation rates in primary B cells infected by different recombinant viruses. While variability in the virus stocks could also account for variations in gene expression, all virus stocks were tested for titers and genome copy number/GIU ratios [56], and only stocks with comparable genome copy#/GIU ratios were used. Therefore, differences in the titers of our virus stocks should not be great enough to account for the effects noted in our experiments.

**Figure 8:**
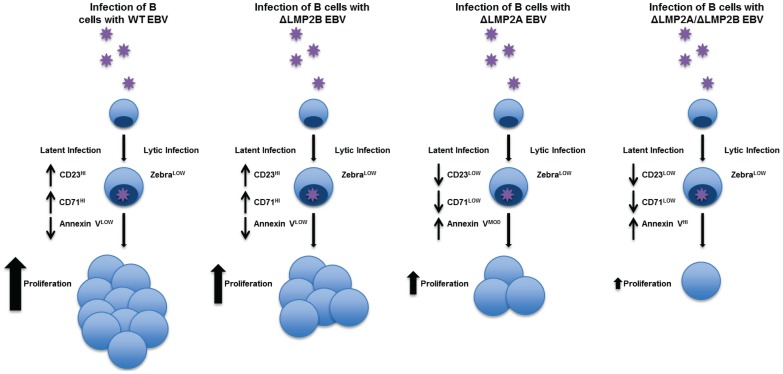
Schematic representation of role for LMP2 in early EBV infection leading to B cell proliferation.

In conclusion, EBV infection leads to activation and proliferation of B cells that result in efficient production of LCLs (Figure 8, Wt panel). Our kinetic analyses show that LMP2A plays critical roles in activation, proliferation, and survival of B cells during the early stages of infection (Δ2A vs wt infection). These effects were not due to loss of stable latency or altered latent gene expression, but appeared to effectively decrease the probability that an LCL would be established (Figure 8, ΔLMP2A panel). LMP2B did not seem to play significant roles in B cell activation, survival, or regulation of gene expression. Although LMP2B-deficient EBV induced lower numbers of proliferating B cells, this virus was as capable of inducing primary B cell activation and establishment of LCLs as wt EBV (Figure 8, ΔLMP2B panel). The loss of both protein isoforms caused the lowest levels of B cell activation, proliferation and survival, which resulted in the lowest probability of establishing LCLs (Figure 8, ΔLMP2A/ΔLMP2B panel). Our studies have confirmed the advantage of LMP2A expression in early B cell infection and LCL formation *in vitro*. The exact mechanisms by which LMP2 elicits these effects on infected B cells require further investigation.
